# Sidewall patterning of organic–inorganic multilayer thin film encapsulation by adhesion lithography

**DOI:** 10.1038/s41598-023-39155-w

**Published:** 2023-07-31

**Authors:** Seung Woo Lee, Heyjin Cho, Choel-Min Jang, Myung-Soo Huh, Sung Min Cho

**Affiliations:** 1 School of Chemical Engineering, Sungkyunkwan University (SKKU), Suwon, 16419 Korea; 2 Samsung Display Co. Ltd., Yongin, 17113 Korea

**Keywords:** Chemical engineering, Materials for devices

## Abstract

A simple sidewall patterning process for organic–inorganic multilayer thin-film encapsulation (TFE) has been proposed and demonstrated. An Al_2_O_3_ thin film grown by atomic layer deposition (ALD) was patterned by adhesion lithography using the difference in interfacial adhesion strength. The difference in interfacial adhesion strength was provided by the vapor-deposited fluoro-octyl-trichloro-silane self-assembled monolayer (SAM) patterns formed by a shadow mask. The sidewall patterning of multilayer TFE was shown possible by repeating the adhesion lithography and the vapor deposition of organic polymer and SAM patterns using shadow masks. The proposed process has the advantage of being able to pattern the blanket ALD-grown Al_2_O_3_ thin films by adhesion lithography using a SAM pattern that can be more accurately predefined with a shadow mask.

## Introduction

For flexible organic light-emitting diode (OLED) displays, an organic–inorganic thin-film encapsulation (TFE) structure that blocks external moisture or oxygen is essential to ensure a long lifetime of organic devices. To effectively prevent moisture permeation to the organic devices, a high-density inorganic thin film with excellent moisture barrier properties is required for the TFE structure. However, since inorganic thin films alone are fragile, organic–inorganic multilayer thin films are commonly used to impart flexibility to the TFE structure^1–11^. This organic–inorganic multilayer encapsulation structure is efficient because it can utilize a repeated multilayer structures to obtain a lower water vapor transmission rate in addition to the advantage of securing flexibility. As the inorganic thin films, silicon nitride^12^ or aluminum oxide^5,6,8,13^ grown by plasma-enhanced chemical vapor deposition (PECVD) or atomic layer deposition (ALD) method is mainly used. On the other hand, as the organic thin films, polymers by wet coating such as inkjet printing^14^ or dry coating such as thermal or plasma polymerization^5,6,15^ are generally utilized.

**Figure 1:**
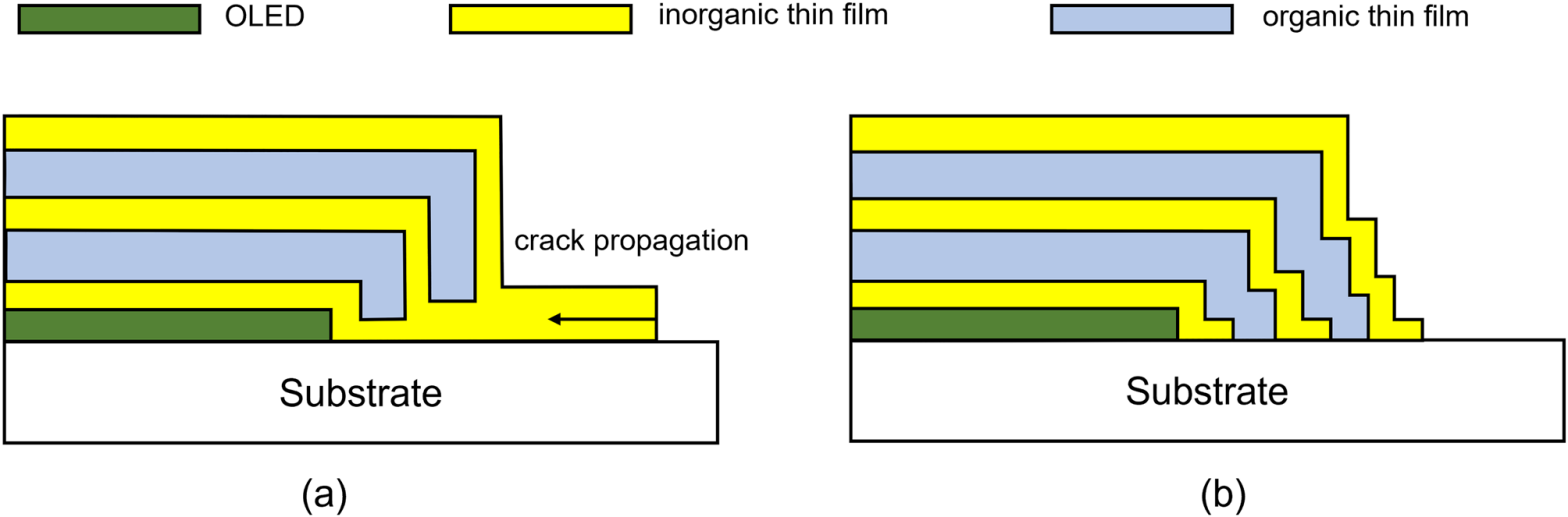
(**a,b**) Two patterned sidewall structures for organic–inorganic multilayer encapsulation.

OLED displays are evolving to become more flexible and have thinner bezels. In this respect, sidewall patterning of organic–inorganic multilayer TFE is becoming increasingly important not only to implement a thin bezel, but also to block moisture ingress through the sides around the TFE structure. Currently, for the sidewall patterning of the TFE, liquid-phase inkjet printing and vapor-phase deposition through a shadow mask are used for organic and inorganic thin films, respectively^3,4,10,14^. However, inkjet printing of organic layers has a disadvantage in that the process speed is slow, and vapor deposition of inorganic layers using a shadow mask also has a disadvantage in that it is difficult to create sharp pattern edges, and thus further process improvement is required. Nevertheless, studies related to TFE have been conducted mainly on moisture-barrier properties^7–9,12,13^ or mechanical deformation stability^5,10,11,15^ of TFE, and little research has been done on sidewall patterning of TFE. In an organic–inorganic multilayer TFE structure, the inorganic thin film blocks moisture penetration, and the organic thin film serves to provide flexibility to the TFE structure. Therefore, to prevent moisture ingress through the sides of the TFE, the inorganic thin films must completely cover the organic thin film patterns at the TFE boundary as shown in Fig. 1a, b. Figure 1a is a TFE structure currently being used in which the inorganic thin film is blanket deposited and only the organic thin film is patterned. On the other hand, Fig. 1b requires a more complicated process because both inorganic and organic thin films need to be patterned. Moreover, patterning of inorganic thin films grown by ALD or PECVD generally requires a photolithography process, so the complexity inevitably increases as the number of layers increases. To reduce this complexity, a simpler patterning method is desirable.

Adhesion lithography is a patterning method that exploits a difference in adhesion strength of a thin film deposited on a patterned substrate with a distinct difference in surface energy. A large difference in surface energy between patterns can be effectively created by pattern-coating a substrate with a material such as self-assembled monolayer (SAM) molecules. When peeling off an inorganic thin film deposited on the SAM-patterned substrate by applying a glue layer thereon, a pattern region with low adhesion strength is torn off but a pattern region with relatively high adhesion strength stays. This lithographic method has been attempted to form a nanogap with a SAM molecular length of 10 nm and has been reported in many research articles^16–18^. The application of this method is effective and very simple for the patterning of uncomplicated structures.

In this study, sidewall patterning of organic–inorganic multilayer TFE was studied using the adhesion lithography method. The TFE is composed of an organic thin film polymerized in plasma using n-hexane precursor and an aluminum oxide (Al_2_O_3_) inorganic thin film deposited by an ALD method. The plasma polymer was pattern-deposited using a shadow mask, while Al_2_O_3_ thin films were blanket-deposited. The Al_2_O_3_ patterns were then formed by the adhesion lithography with SAM patterns which were pattern-deposited using a shadow mask. These two organic and inorganic thin films were alternately stacked to form an organic–inorganic multilayer encapsulation structure. As shown in Fig. 1a, even when only the organic thin films are patterned and the Al_2_O_3_ thin films are not patterned, moisture ingress from the sides of the TFE can be prevented. However, if all the inorganic thin films are connected as in the case of Fig. 1a, there is a concern that cracks at panel edges, which may occur in the process of cutting the OLED panels after completion of the TFE process, may gradually propagate toward the panel and affect the entire TFE. On the other hand, a TFE structure like Fig. 1b, in which both organic and inorganic thin films are patterned, has an advantage in that cracks that may occur at the edges can be limited to the outermost inorganic layer. Therefore, in this study, a process for forming the TFE structure as shown in Fig. 1b was proposed, and the result of this study is expected to be utilized in the actual TFE process of OLED display panels. In particular, it is important because it is a method for obtaining a TFE structure that is simple but stable against deformation without using a process such as a wet process or photolithography. In this study, in order to verify the effectiveness of the encapsulation multilayer structure with patterned sidewalls, the lateral propagation of cracks was experimentally observed when folding deformation was applied to the Al_2_O_3_ layer. It was also experimentally confirmed that the propagation of cracks along the Al_2_O_3_ layers could be prevented by spatially separating the layers. Therefore, it can be expected that the encapsulation structure in which the sidewalls are patterned by separating the Al_2_O_3_ layers is more stable against folding deformation than the structure in which the layers are connected, and this expectation was experimentally verified in this study.

## Experimental

As a substrate for the TFE, a polyethylene terephthalate (PET) film cut into 1 inch in width and length were used. In order to measure the cross-sectional image of the fabricated TFE, a silicon wafer of the same size was used instead of the PET substrate. The substrates were first cleaned with acetone, methanol, and deionized water. A patterned metal shadow mask was placed on the substrate as close as possible using a magnet. The substrate attached with the shadow mask was fixed upside down on the top of a container with fluoro-octyl-trichloro-silane (FOTS) SAM, and the SAM was vapor-deposited for 10 h in a vacuum oven preheated to 80 °C. The SAM was deposited upwards onto the substrate through the open area of the shadow mask to form a SAM pattern. After the patterning of the Al_2_O_3_ layer by adhesion lithography was completed, the remaining SAM pattern was removed by treatment with ultraviolet (UV)-ozone for 20 min.

Al_2_O_3_ thin films were grown on the SAM-patterned substrate by an ALD method. The base pressure of the ALD chamber was 0.002 torr and the substrate temperature was maintained at 100 °C during the deposition. TMA (trimethy aluminum) and ozone were used as precursors for aluminum and oxygen, respectively, and both injection times were fixed at 2 s. After each precursor injection cycle, the chamber was purged with argon for 20 s. In this deposition condition, the average deposition thickness per cycle was 1 Å.

For adhesion lithography, a UV curable resin (NOA61, Norland, USA) was thinly spread on the Al_2_O_3_-deposited substrate using a roller. The resin was cured for 185 s using a UV exposure apparatus. The cured resin was then peeled off so that the Al_2_O_3_ layer deposited on the SAM surface was selectively removed. For the organic–inorganic multilayer TFE, the organic thin film was deposited via plasma polymerization by introducing *n*-hexane vapor into a PECVD chamber. This plasma polymer was patterned using a shadow mask. The deposition was carried out by injecting argon (30 sccm) and n-hexane (20 sccm) in a plasma of 50 W power, and the deposition rate was 15 nm/min.

A scanning electron microscopy (SEM, XL 30 ESEM-FEG, Philips) was used for the surface analysis and a focused ion beam SEM (LYRA3 GMH, TESCAN) was used for cross-sectional analysis. For composition analysis, X-ray photoelectron spectroscopy (XPS, Thermo Scientific) and energy dispersive X-ray spectroscopy (EDAX) were used.

## Results and discussion

**Figure 2:**
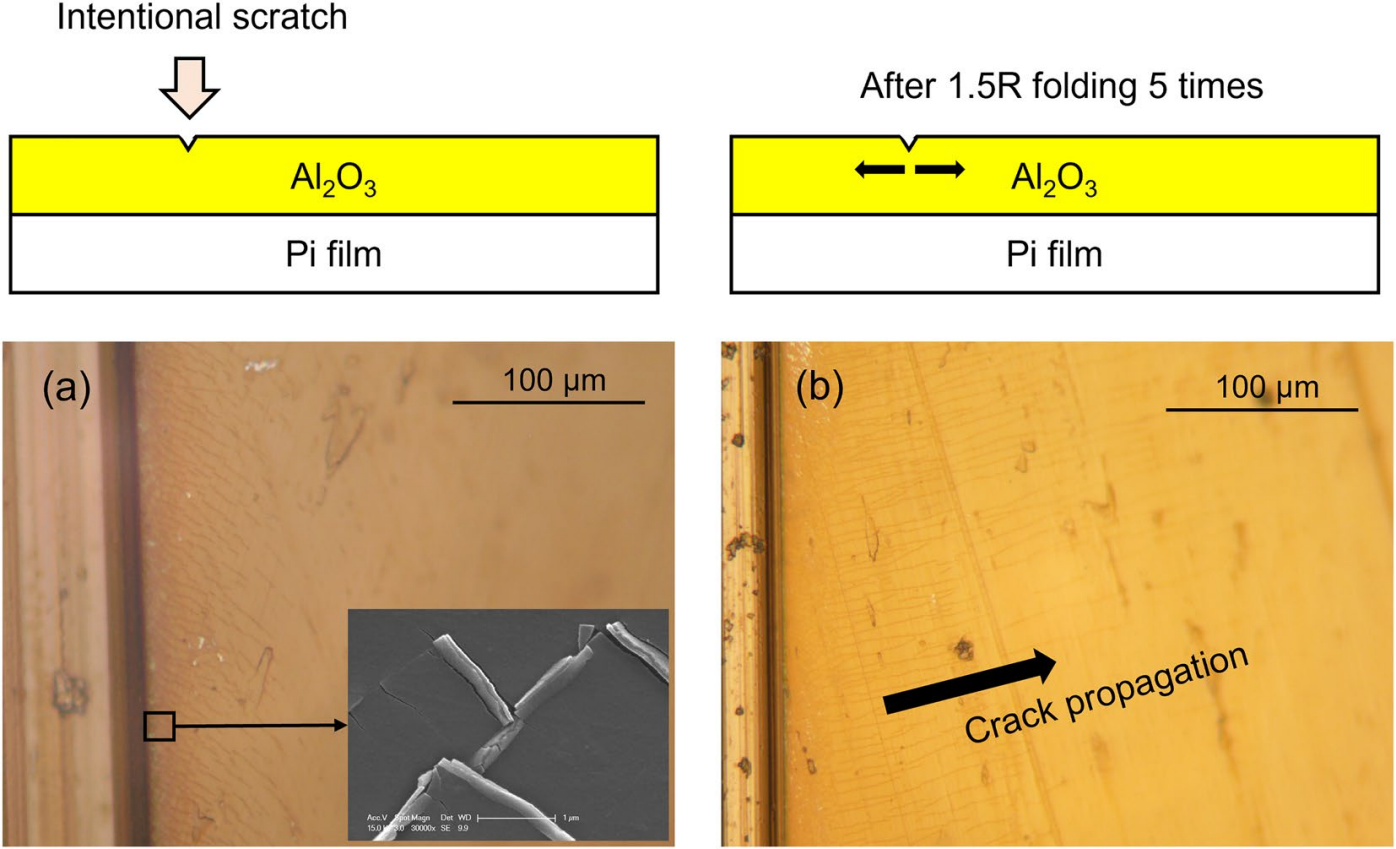
(**a**) Creation of cracks by intentional scratching on the surface of Al_2_O_3_ thin film, (**b**) propagation of cracks along the surface of the Al_2_O_3_ thin film after repeated folding 5 times under 1.5R condition.

**Figure 3:**
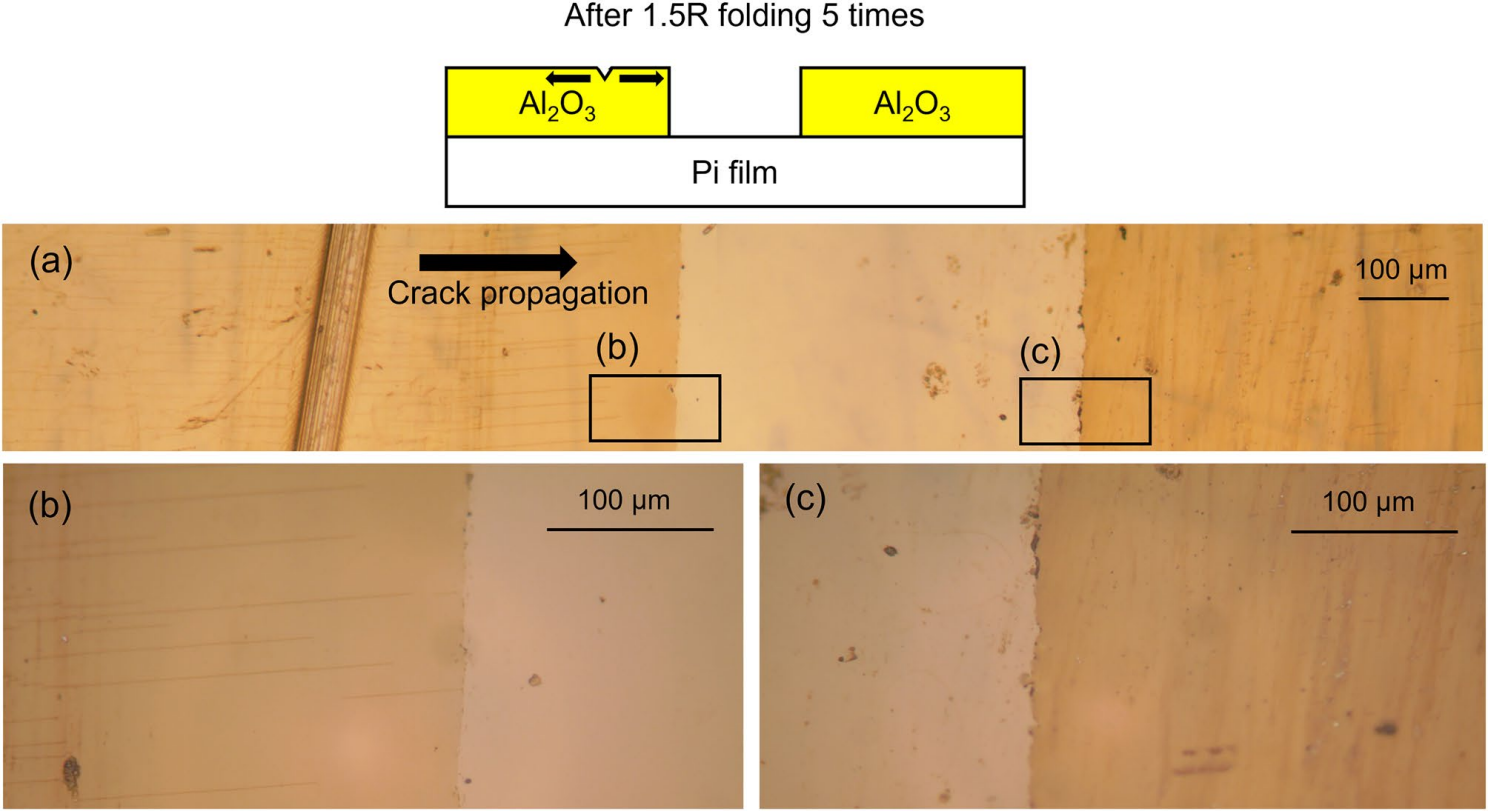
Crack propagation after 5 times of repeated folding under 1.5R condition along Al_2_O_3_ thin film divided into two regions.

To protect OLED devices by using an organic–inorganic multilayer TFE structure, the inorganic thin film must completely cover the organic thin film as shown in Fig. 1a or b. These TFE structures require patterning of organic and/or inorganic thin films. As shown in Fig. 1a, when all inorganic thin films are connected, cracks that may occur at the TFE boundary can affect the entire TFE. The cracks may gradually propagate over time toward the device and eventually lead to TFE damage, allowing moisture to penetrate the device through the cracks. After the TFE process is completed, in the process of cutting the perimeter of the panel, if there is an Al_2_O_3_ layer at the top surface, cracks can occur in the Al_2_O_3_ layer at the perimeter as shown in Fig. 2a. Figure 2a shows that very fine cracks occur around the deliberately scratched area. As in this case, when cracks occur on the Al_2_O_3_ surface in the process of cutting the panel, the possibility of the cracks propagating toward the panel increases when repeated folding of the display panel is applied. As shown in Fig. 2b, it can be confirmed that the cracks formed due to intentional scratches propagate in the orthogonal direction of folding after folding 5 times with a radius of 1.5 mm (1.5R). So, it is desirable to avoid cracks that exist at the perimeter because there is a risk of continuing propagation toward the panel over time. However, since the occurrence of cracks cannot be completely excluded during the panel cutting process, it is advantageous to prevent crack propagation along the rigid Al_2_O_3_ thin films. To confirm this experimentally, crack propagation was observed when the inorganic Al_2_O_3_ thin film was divided into two regions. As shown in Fig. 3, after intentionally forming cracks in one of the two regions, folding was performed 5 times under the condition of 1.5R. As described above, the cracks continue to propagate and proceed to the interface where the Al_2_O_3_ thin film is removed (Fig. 3a, b). However, no cracks were found in the Al_2_O_3_ thin film on the other side (Fig. 3c). From this result, it can be confirmed that when the inorganic Al_2_O_3_ thin films are separated from each other, the crack propagation occurs confined to each Al_2_O_3_ thin film. For this reason, it is expected that the TFE structure as shown in Fig. 1b will be more effective in preventing moisture penetration in situations where repeated folding occurs.

In the conventional TFE process, the organic thin film is patterned directly by inkjet printing, and the inorganic thin film is patterned by vapor deposition methods such as ALD or PECVD using shadow masks. When the inorganic thin film is deposited by ALD through a shadow mask, unwanted deposition of the inorganic thin film (so-called tailing) occurs underneath the shadow mask as the deposition precursors sneak into the space between the shadow mask and the substrate. This tailing phenomenon is more severe in ALD, where the deposition rate is very slow. As shown in Fig. S1, the tailing of Al_2_O_3_ thin films grown by ALD reaches more than 15 mm below the shadow mask, and the thicker the Al_2_O_3_ thin film, the more severe the tailing phenomenon. If the tailing is severe, the Al_2_O_3_ thin film cannot be patterned as desired with the shadow mask, and eventually cracks may occur on the Al_2_O_3_ surface in the process of cutting around the panel for a thin bezel display. In addition, there is a concern that the entire TFE may be affect by the propagation of these cracks, especially when all Al_2_O_3_ thin films are connected as shown in Fig. 1a. To lessen this problem, accurate patterning of the inorganic Al_2_O_3_ thin film is essential. Photolithography may be considered as the patterning method for the inorganic thin film, but it is not preferred in consideration of the process complexity and cost. Instead, in this study, the inorganic thin film was patterned by adhesion lithography using FOTS SAM.

First, ALD of Al_2_O_3_ thin films on the FOTS SAM surface was investigated. ALD is highly dependent on the surface chemical properties because it occurs via the surface adsorption of the reactant precursors. Therefore, it can be expected that the ALD of the Al_2_O_3_ thin film will proceed differently on the silicon substrate and the SAM surfaces. Area-selective deposition in which ALD occurs only in a designated area by using the difference in surface characteristics has been studied extensively in recent years^19–22^. If such area-selective ALD is possible, the Al_2_O_3_ thin film patterns can be easily produced without a separate lithography process. So, in this study, an experiment was first conducted to find out whether the Al_2_O_3_ thin film can be selectively grown by ALD on the silicon surface patterned with FOTS SAM. The adsorption characteristics of the TMA precursor for the ALD will differ significantly between the silicon substrate and FOTS SAM surfaces, where hydroxyl groups and fluorine atoms are exposed, respectively. The water contact angles of the bare silicon and FOTS SAM surfaces were about 48° and 105°, respectively. During the ALD cycle, the change in the water contact angle was measured to confirm the deposition selectivity of the Al_2_O_3_ thin film for each surface. Since ALD of Al_2_O_3_ thin films occurs well on the silicon surface, the water contact angle starts to decrease from the beginning as the ALD cycle is repeated. This is because the water contact angle on the Al_2_O_3_ surface is much lower than that on the bare silicon surface. When the Al_2_O_3_ ALD cycle is repeated at a temperature of 90 °C on the FOTS SAM coated surface, the water contact angle also continuously decreased as shown in Fig. S2a. This means that an Al_2_O_3_ thin film is growing on the FOTS SAM surface. If the water contact angle of the FOTS SAM surface remain unreduced during the ALD cycles, it can be understood that the Al_2_O_3_ thin film is not growing on this surface. When the substrate temperature was increased to 100 °C, the water contact angle on the FOTS SAM surface was maintained for about the initial 35 ALD cycles, and then rapidly decreased as the cycles were repeated (Fig. S2b). It is interpreted that this is because Al_2_O_3_ growth is delayed on the FOTS SAM surface due to the inhibition of TMA precursor adsorption at the higher substrate temperature. However, it was difficult to increase the number of these initial delay cycles even if the injection or purge time of the precursors is adjusted and optimized. At higher substrate temperatures, more selective Al_2_O_3_ ALD may be possible, but since the TFE process temperature for OLED displays is limited to 100 °C, higher temperatures were not investigated in this study. The average deposition rate shown together in the Fig. S2 is calculated from the thickness of Al_2_O_3_ deposited on the silicon surface. As expected from this experiment, the selective ALD was limited to only the initial 35 cycles at 100 °C, and Al_2_O_3_ ALD eventually occurred on the SAM surface after the initial delay cycles. In fact, when an Al_2_O_3_ thin film grown for 1,500 ALD cycles on the silicon substrate patterned with the SAM, the deposition selectivity on each surface could not be confirmed as shown in Fig. S3. Both the silicon substrate surface and the SAM surface showed almost the same Al_2_O_3_ thickness of 160 nm after the ALD. It means that the blanket ALD of Al_2_O_3_ thin film on the silicon substrate patterned with FOTS SAM is possible if the Al_2_O_3_ thickness to be grown is sufficiently thick.

**Figure 4:**
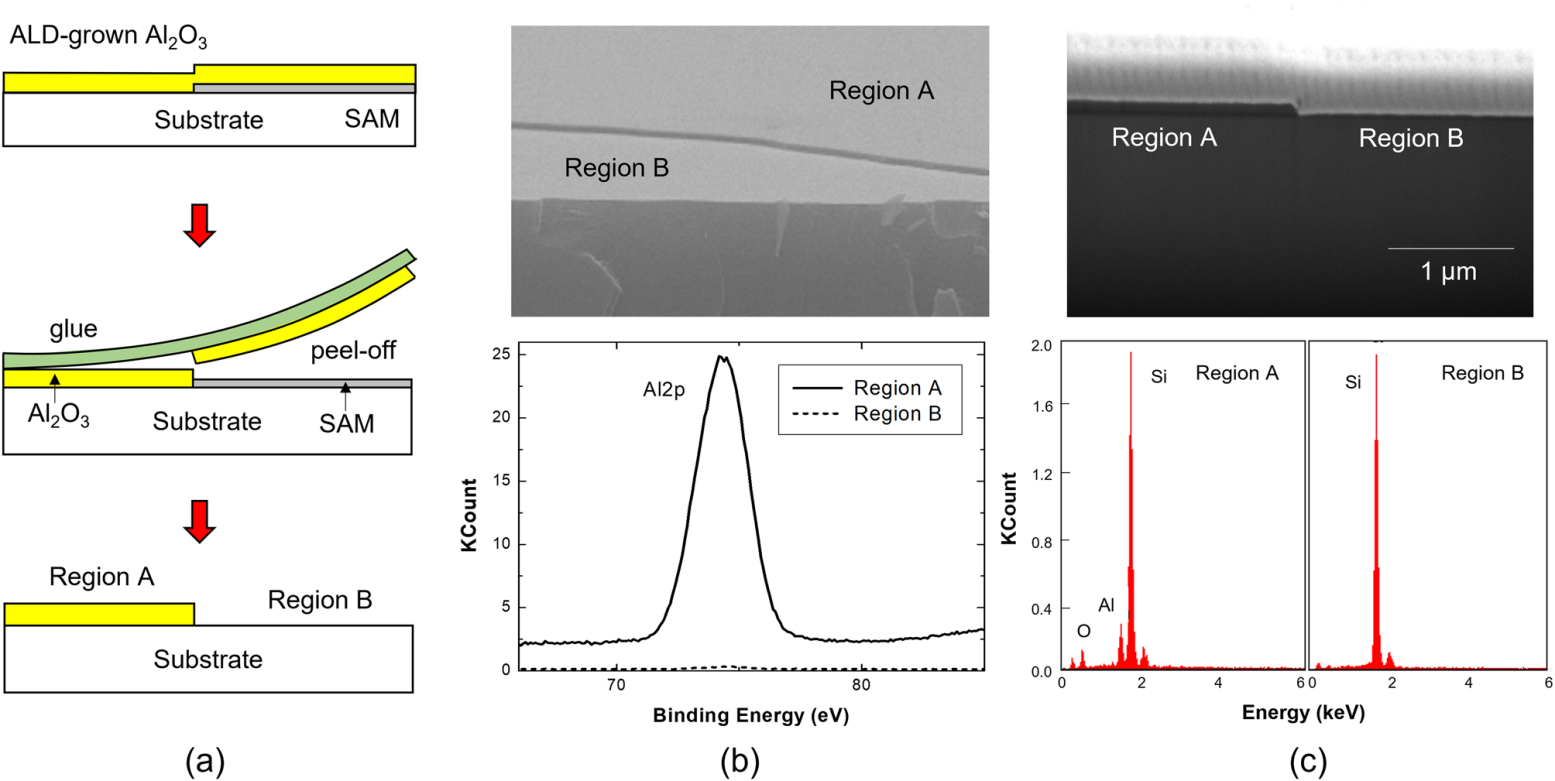
(**a**) Schematic diagram of adhesion lithography process for Al_2_O_3_ pattern formation; (**b,c**) SEM images of the produced Al_2_O_3_ patterns, and XPS and EDAX analyses results showing that Al was not detected in region B where Al_2_O_3_ was peeled off.

The Al_2_O_3_ thin film grown by the blanket ALD was patterned by adhesion lithography. The FOTS SAM was first pattern-deposited on the substrate surface using a shadow mask. After depositing an Al_2_O_3_ thin film on the SAM-patterned substrate, the Al_2_O_3_ thin film on the SAM-coated area was selectively removed using a UV-curable glue layer as shown in Fig. 4a. Unlike Al_2_O_3_ ALD, the SAM pattern, which is vapor-coated through a shadow mask, has a sharp pattern boundary without tailing, as shown in the SEM images in Fig. 4b, c. Using this adhesion lithography, the Al_2_O_3_ thin film was accurately patterned according to the pattern defined by SAM. As a result of XPS and EDAX analyses of Al_2_O_3_ patterns obtained by the adhesion lithography, it was confirmed that there were no Al_2_O_3_ residues in the area where Al_2_O_3_ is peeled off.

This adhesion lithography is possible because of the difference in adhesion strength between the two interfaces of silicon substrate-Al_2_O_3_ thin film and FOTS SAM surface-Al_2_O_3_ thin film. Table S1 shows the contact angle and surface energy of silicon, FOTS SAM, and Al_2_O_3_, obtained using polar and non-polar solvents. Using these values and the equation below, the work of adhesion for the two interfaces of silicon substrate-Al_2_O_3_ thin film and FOTS SAM surface-Al_2_O_3_ thin film was calculated as 128.2 and 43.03 mJ/m^2^, respectively. Due to such a large difference in the work of adhesion, the Al_2_O_3_ thin film on the surface of the FOTS SAM is selectively peeled off by the glue layer.

$${W}_{{1,2}}=4\left[\left(\frac{{\gamma }_{1}^{Dispersive}\cdot{\gamma }_{2}^{Dispersive}}{{\gamma }_{1}^{Dispersive}+{\gamma }_{2}^{Dispersive}}\right)+\left(\frac{{\gamma }_{1}^{Polar}\cdot{\gamma }_{2}^{Polar}}{{\gamma }_{1}^{Polar}+{\gamma }_{2}^{Polar}}\right)\right]$$

**Figure 5:**
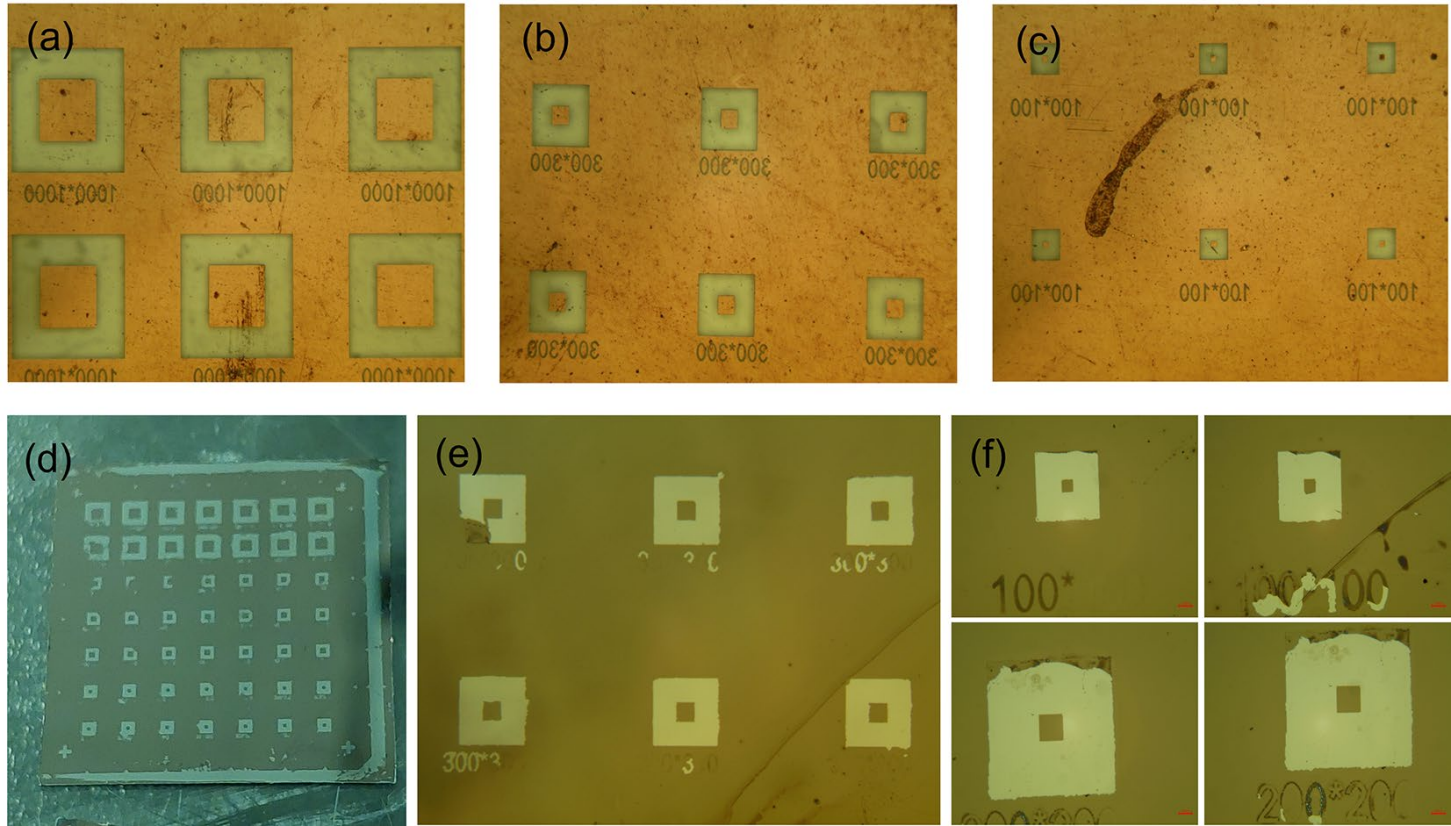
(**a**) 1000 μm × 1000 μm, (**b**) 300 μm × 300 μm, and (**c**) 100 μm × 100 μm photomask pattern images; (**d–f**) optical microscope images of Al_2_O_3_ patterns obtained by adhesion lithography.

To confirm the resolution limit of adhesion lithography, fine SAM patterns were formed using various square photomask patterns ranging in size from 1000 μm × 1000 μm to 100 μm × 100 μm as shown in Fig. 5a–c. Since this experiment is intended to measure the resolution limit in the peel-off process of the Al_2_O_3_ thin film, the SAM was patterned using a photomask capable of patterning with higher resolution instead of using a shadow mask. The photolithographic process was performed so that the SAM pattern could be formed in the bright green area on the photomask shown in Fig. 5a–c. After blanket ALD growth of an Al_2_O_3_ thin film on the top of the SAM patterns, peel-off with a glue layer can lift-off the Al_2_O_3_ thin film on the SAM pattern area as shown in Fig. 5d–f. In these figures, the bright area is the area where Al_2_O_3_ is lifted off, and it is possible to form Al_2_O_3_ patterns as small as 100 μm × 100 μm in size.

**Figure 6:**
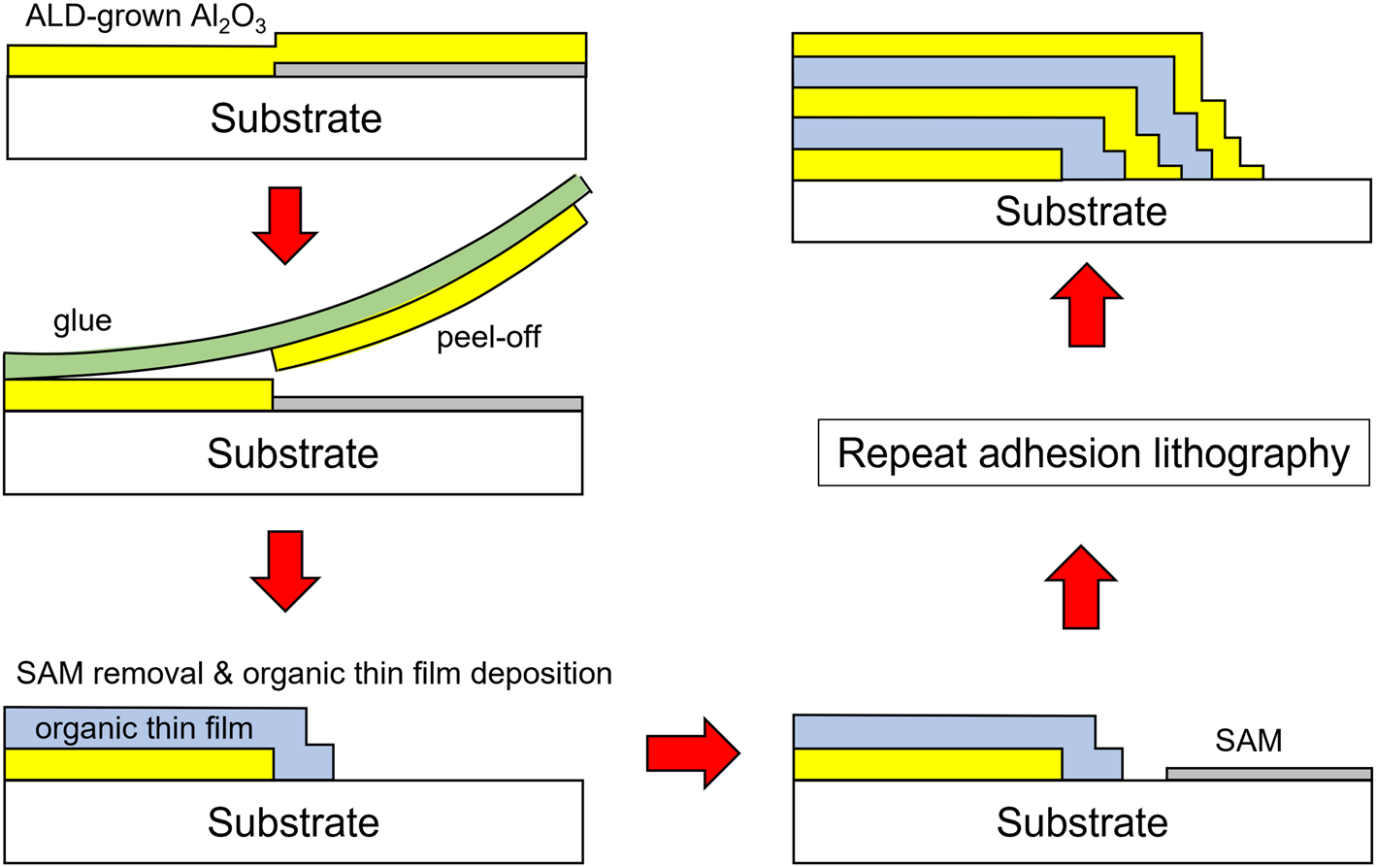
Schematic diagram of the process sequence for sidewall patterning of organic–inorganic multilayer encapsulation.

An adhesion lithography process for forming an organic–inorganic multilayer TFE structure as shown in Fig. 1b was proposed and attempted to demonstrate experimentally. This process is schematically shown in Fig. 6. The first Al_2_O_3_ thin film was patterned on the substrate by adhesion lithography using FOTS SAM. A plasma polymer layer was deposited on the patterned Al_2_O_3_ thin film using a shadow mask. The plasma polymer layer to be deposited also has a tailing of about tens of microns underneath the shadow mask, but the degree of tailing is much less than that of Al_2_O_3_ ALD, which shows a very slow deposition rate. After deposition of the plasma polymer pattern, the second SAM layer is patterned using a shadow mask on the outside of the polymer pattern. Then, a second Al_2_O_3_ layer is blanket-deposited to cover the entire surface and adhesion lithography is performed in the same manner as before to form the predefined Al_2_O_3_ pattern by SAM. By repeating this process, it is possible to accomplish the sidewall patterns of the organic–inorganic multilayer TFE structure.

**Figure 7:**
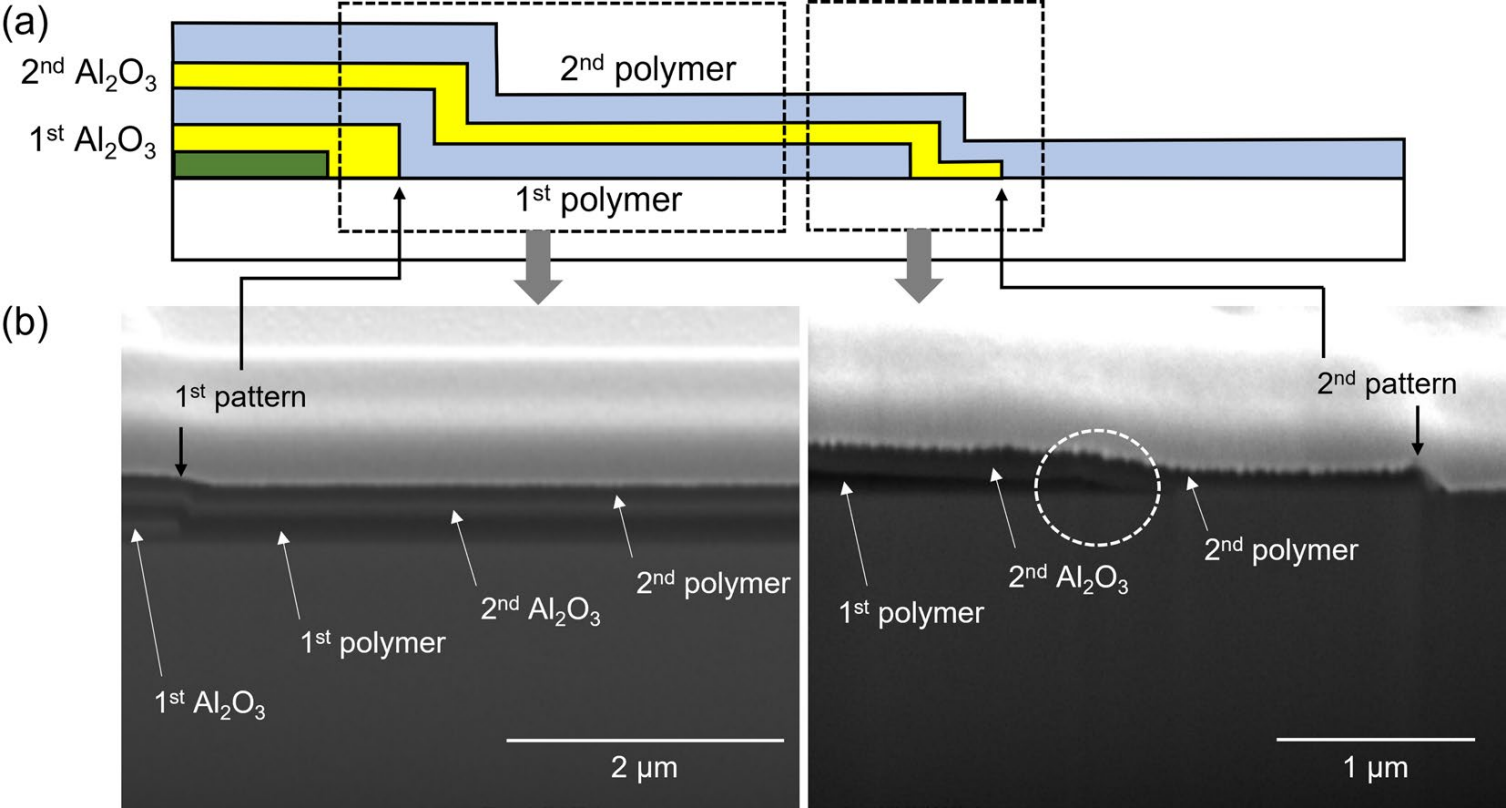
(**a**) Schematic cross-sectional diagram and (**b**) SEM images of sidewall structure of organic–inorganic multilayer encapsulation produced by adhesion-lithography patterning of Al_2_O_3_ thin films and shadow-mask patterning of polymer thin films.

**Figure 8:**
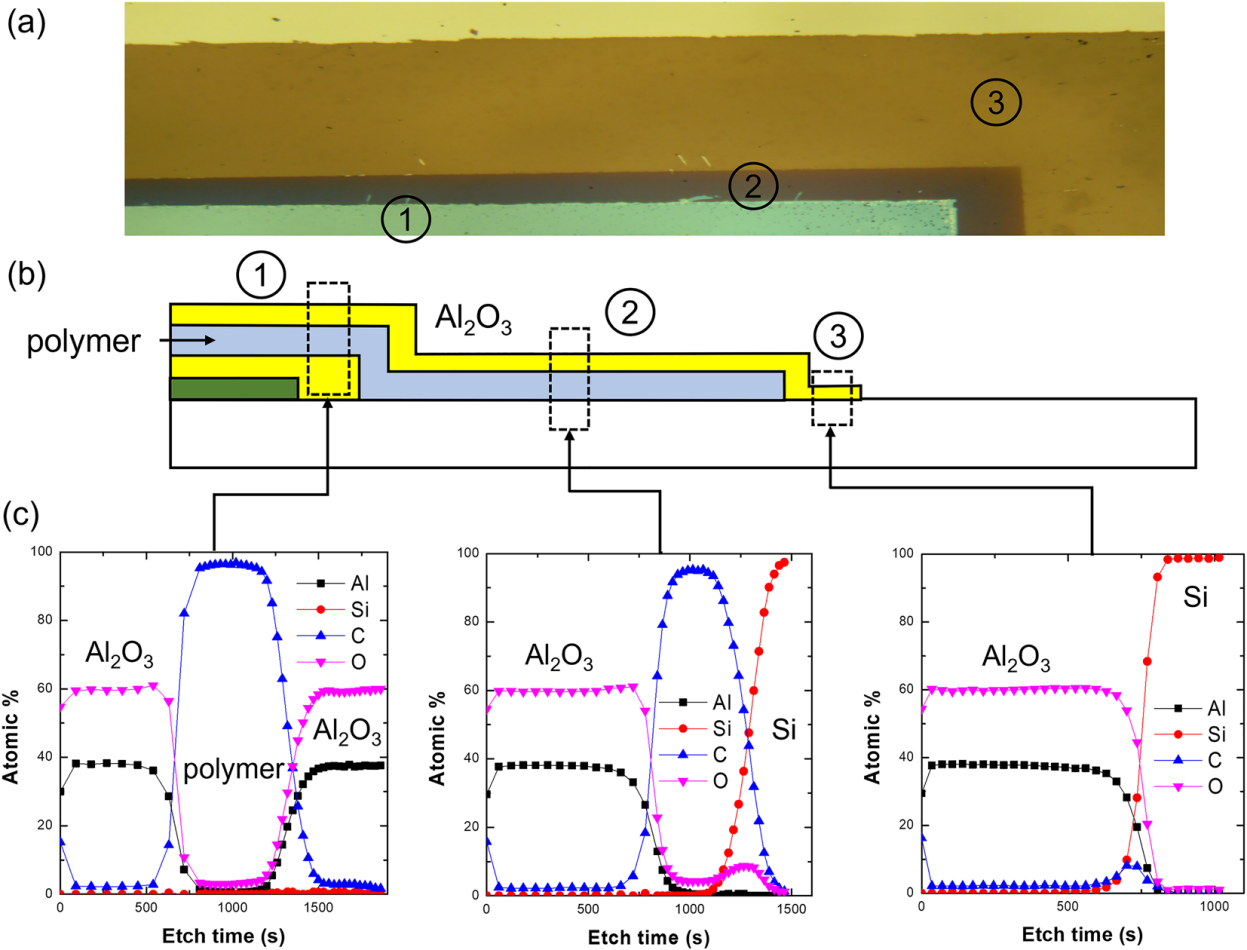
(**a**) Top-view optical microscopic image, (**b**) schematic cross-sectional diagram, and (**c**) XPS depth profiles in each area of organic–inorganic multilayer encapsulation produced by adhesion-lithography patterning of Al_2_O_3_ thin films and shadow-mask patterning of polymer thin films.

Figure 7 shows the sidewall structure of organic–inorganic multilayer TFE fabricated according to the proposed scheme. This TFE is composed of two layers of Al_2_O_3_ thin film and two layers of polymer thin film. As shown in the figure, the first and second pattern edges of the Al_2_O_3_ thin film are sharply defined by adhesion lithography described above. The first polymer thin film was patterned using a shadow mask. From the dotted circle in the SEM image of Fig. 7, it can be confirmed that the tailing that occurred when the first polymer thin film was patterned as a shadow mask was not so severe. From the SEM image in Fig. 7, it was found that organic–inorganic multilayer encapsulation is possible by the proposed adhesion lithography. However, the SEM image is somewhat blurry, so it is difficult to visually confirm clearly. In order to clearly confirm the encapsulation structure through component analysis, an encapsulation structure with two layers of Al_2_O_3_ thin film and one layer of polymer thin film was fabricated. A top-view optical micrograph of the sidewall patterned panel edges is shown in Fig. 8. Circled numbers indicate each area where the pattern was formed, and the area corresponding to the circle number is indicated in the cross-sectional schematic of the structure. Each area was verified with the XPS depth profile measurement to confirm that the desired thin film layer was formed in the depth direction. As shown in Fig. 8, it was confirmed that each area had the desired layer structure. However, as shown in Figs. 7 and 8, the two layers of Al_2_O_3_ thin film not only completely cover the display area but are also separated from each other. In this study, the processes utilized for sidewall patterning of the multilayer TFE structure were ALD of Al_2_O_3_ layer, vapor phase plasma polymerization of polymer layer, and vapor deposition of SAM pattern for adhesion lithography. Since the fabrication of the sidewall structure proceeds only through vapor deposition and adhesion lithography processes, there are advantages in terms of process continuity and simplicity compared to the case of using photolithography. In the process proposed in the study, blanket ALD is used for Al_2_O_3_ thin film, which is difficult to pattern with a shadow mask, and instead, the Al_2_O_3_ thin film is patterned by adhesion lithography using the FOTS SAM, which is easier to pattern with a shadow mask. The proposed process is expected to be utilized as a simpler multilayer TFE process for thin bezel OLED displays.

Finally, an additional experiment was conducted to verify that the sidewall encapsulation formed of separated Al_2_O_3_ layers is more stable against folding deformation than the case of connected Al_2_O_3_ layers. First, as shown in Fig. S4, a structure in which two Al_2_O_3_ encapsulation layers are connected similarly to Fig. 1a was fabricated on a PET substrate, and scratches were deliberately formed on the side of the structure. In these structures, both the Al_2_O_3_ and polymer layers had a thickness of 50 nm. No cracks were found on the surface of the Al_2_O_3_ layer before folding deformation as shown in Fig. S4. However, after folding five times with a radius of 1.5R, it was confirmed that cracks occurred on all Al_2_O_3_ surfaces of the 2.5 cm × 2.5 cm structure. The cracks on the surface shown in Fig. S4 are generated from both the upper and lower Al_2_O_3_ layers. For comparison, the same experiment was conducted on an encapsulation structure of the same size in which two Al_2_O_3_ layers were separated. This structure is shown in Fig. S5. As in the previous case, no cracks could be identified on the surface of the Al_2_O_3_ layer before folding deformation. From this fact, it can be confirmed that cracks do not occur in the Al_2_O_3_ layers due to peeling stress or pressure applied during the adhesion lithography process. In this case as well, cracks occurred on the Al_2_O_3_ surface after the same folding deformation. In both cases shown in Figs. S4 and S5, cracks in the upper Al_2_O_3_ layer can be identified from the photographs included in the figures, but cracks in the lower Al_2_O_3_ layer cannot be directly verified. However, considering that the crack density is denser in the case of Fig. S4 than in the case of Fig. S5, it can be inferred that cracks also occurred in the lower Al_2_O_3_ layer in the structure shown in Fig. S4. As confirmed in Fig. S5, the density of cracks in the area of the two Al_2_O_3_ layers was much less dense overall, and cracks occurred intensively only at the boundary of the lower Al_2_O_3_ layer. From this fact, it can be seen that in the case of the structure in which the Al_2_O_3_ layers are separated, the crack propagation is limited compared to the case where the layers are connected, and even if new cracks are generated by folding deformation, it occurs intensively near the boundary of the lower Al_2_O_3_ layer, which can receive the greatest stress. This experiment confirms that the encapsulation structure in which the Al_2_O_3_ layers are separated is less prone to cracking than the structure in which these layers are connected. Therefore, it can be expected that the encapsulation shown in Fig. S5 is more stable against folding deformation than the structue in Fig. S4.

After applying the same folding deformation to the two structures shown in Figs. S4 and S5, an optical calcium (Ca) test was performed to compare the encapsulation characteristics. This experiment is to verify which structure is more volnerable to cracking and moisture permeation due to folding deformation. First, a 1.5 cm × 1 cm 200 nm-thick Ca layer was deposited on 5 cm × 5 cm glass plate. Separately, cracks were generated by applying the same folding deformation as in the previous experiment to the PET substrate on which the two encapsulation structures were formed. These substrates were covered on the Ca pattern, and the surroundings were tightly sealed with UV curing resin. The Ca test samples were stored in a chamber at a temperature of 85 °C and a relative humidity of 85%, and the degree of Ca oxidation was measured every 30 min. As expected from the previous experiment, the structure shown in Fig. S5 showed better encapsulation properties under the same conditions. This result is shown in Fig. S6. When the Al_2_O_3_ layers were connected, more than half of the Ca was oxidized after 90 min, whereas when the Al_2_O_3_ layer were separated, there was little change in Ca. This experiment shows that the case of sidewall patterning by separating the Al_2_O_3_ encapsulation layers is more stable against folding deformation, and eventually the encapsulation characteristics are maintained for a longer period of time compared to the case where the Al_2_O_3_ layers were connected.

## Conclusions

In this study, a sidewall patterning process for organic–inorganic multilayer TFE has been proposed. The ALD-grown Al_2_O_3_ thin film was patterned by adhesion lithography using the difference in interfacial adhesion strength which was provided by using a vapor-deposited FOTS SAM through a shadow mask. Since the adhesion strength of the Al_2_O_3_ thin film grown on the FOTS SAM surface was much lower than that of the case grown on silicon, it was possible to easily pattern the Al_2_O_3_ thin film by adhesion lithograph. Sidewall patterning of multilayer TFE was shown possible by repeating the adhesion lithography and the vapor deposition of organic polymer and SAM through shadow masks. The proposed process has the advantage of being able to pattern the blanket ALD-grown Al_2_O_3_ thin films by adhesion lithography using a SAM pattern that can be more accurately predefined with a shadow mask.

## Supplementary Information

Supplementary Information.

## Data Availability

The data that support the findings of this study are available from the corresponding author upon reasonable request.
