# Supplementary material for: Sidewall patterning of organic–inorganic multilayer thin film encapsulation by adhesion lithography
Source: Sci Rep. 2023 Jul 31;13:12394. doi: 10.1038/s41598-023-39155-w (PMC10390510; doi:10.1038/s41598-023-39155-w)
Supplement: Supplementary file 1 — Supplementary Information. [file 41598_2023_39155_MOESM1_ESM.docx]

**Supplementary Information**

Sidewall Patterning of Organic-Inorganic Multilayer Thin Film Encapsulation by Adhesion Lithography

Seung Woo Lee^1^, Heyjin Cho^1^, Choel-Min Jang^2^, Myung-Soo Huh^2^ & Sung Min Cho^1*^

^1^School of Chemical Engineering, Sungkyunkwan University (SKKU), Suwon 16419, Korea.^2^Samsung Display Co. Ltd, Yongin 17113, Korea.


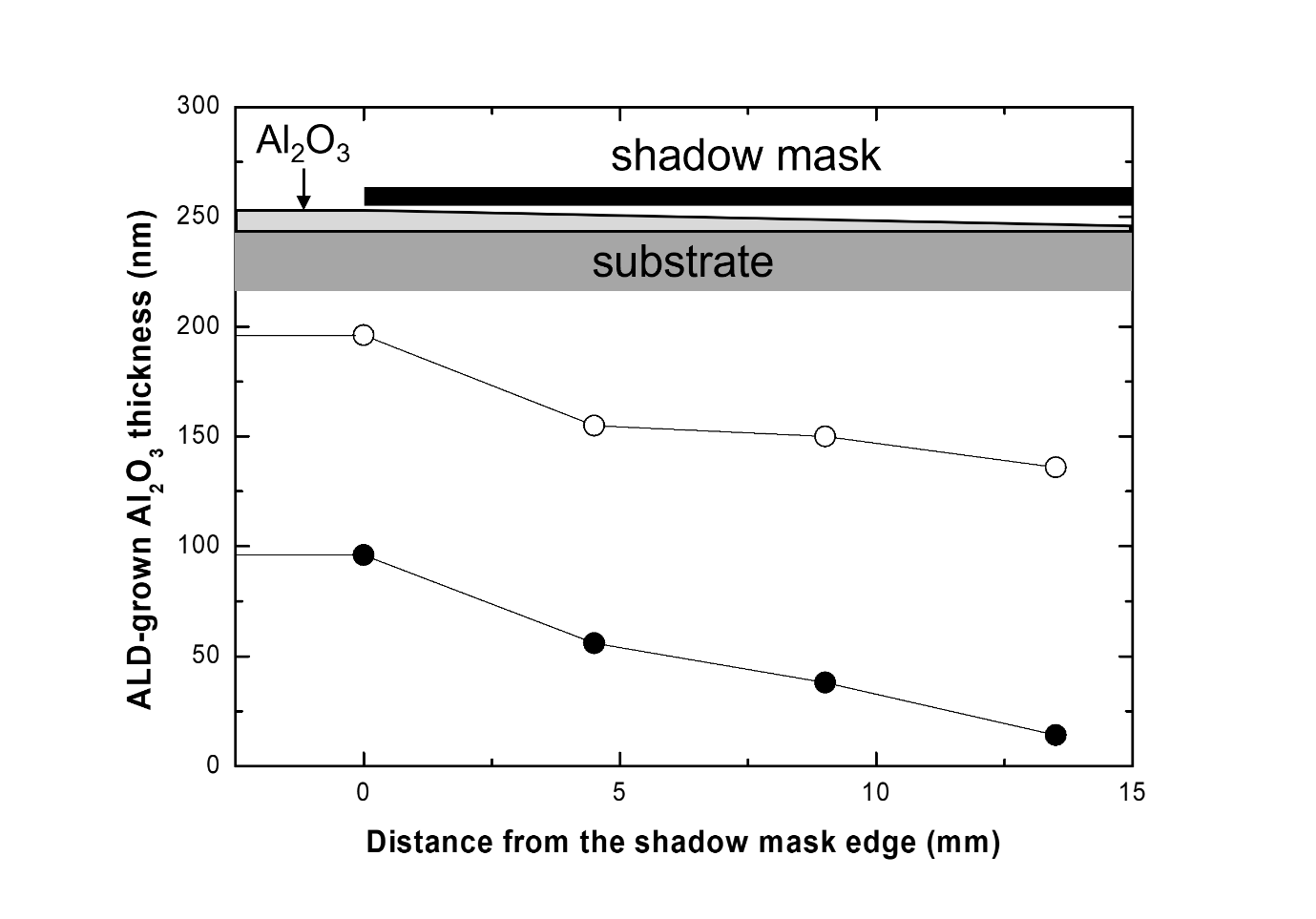


**Figure S1**. Variation in thickness of ALD-deposited Al_2_O_3_ thin film underneath a shadow mask represented as a function of the distance from the shadow mask edge.


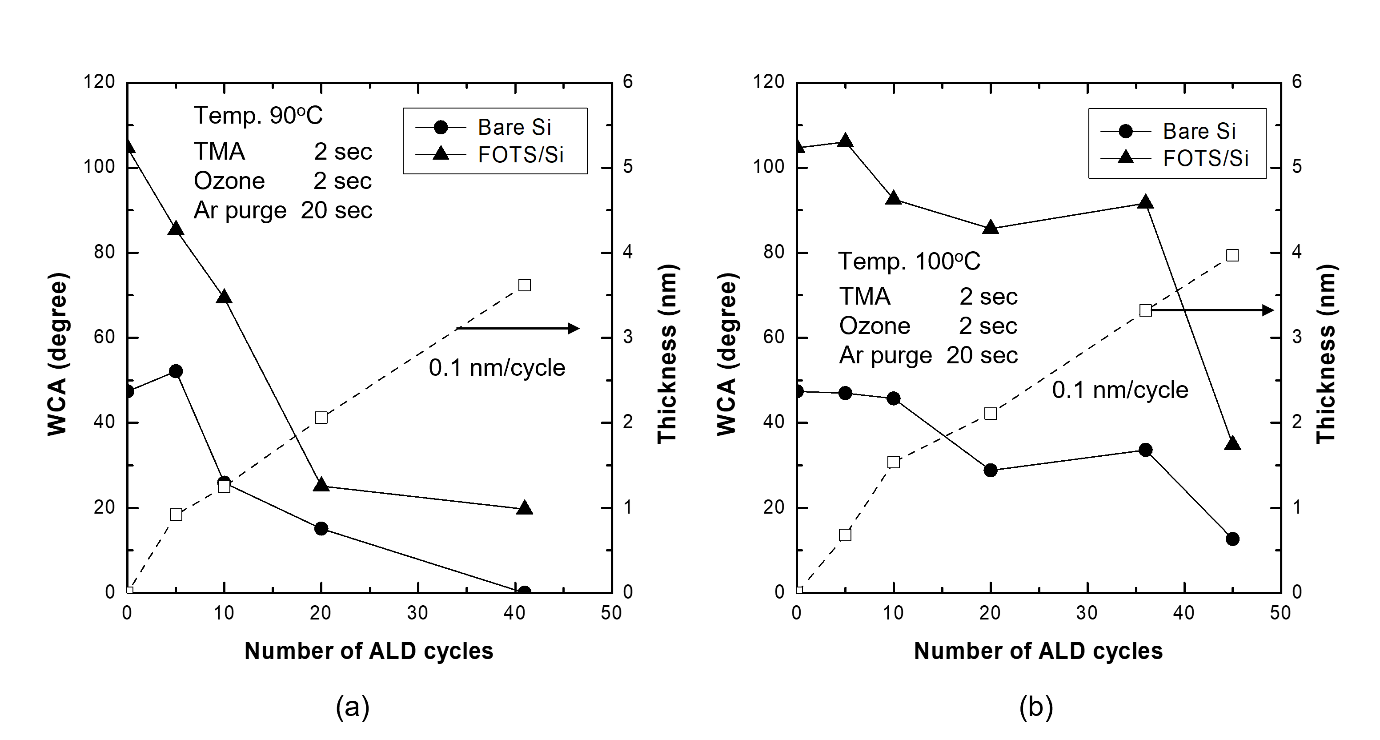


**Figure S2**. Changes in water contact angle of silicon and FOTS SAM surfaces with increasing Al_2_O_3_ ALD cycle at a substrate temperature of (a) 90^o^C and (b) 100^o^C. The dashed line shows the increase in the Al_2_O_3_ thickness on the silicon surface as the ALD cycle increases.


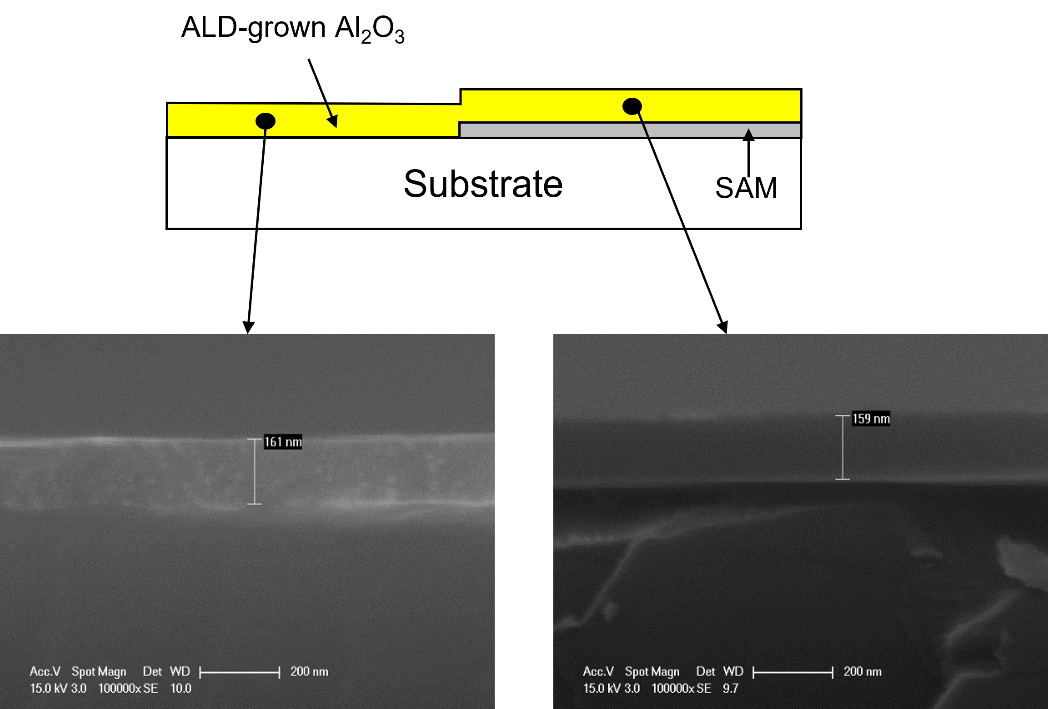


**Figure S3**. Thickness of the Al_2_O_3_ thin film grown with 1,500 ALD cycles on a silicon substrate patterned with FOTS SAM.


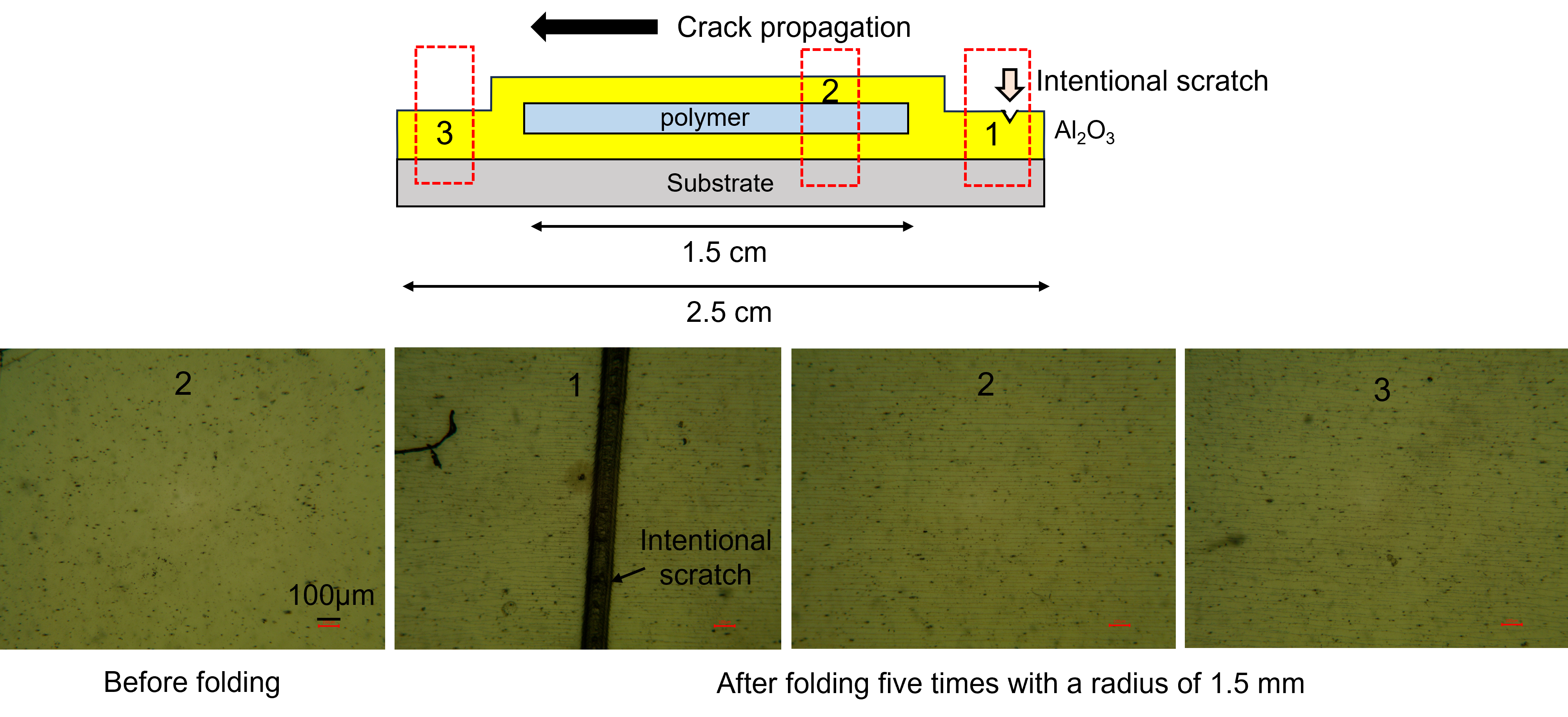


**Figure S4**. Cross-sectional schematic of the encapsulation structure in which two Al_2_O_3_ layers are connected, and surface photographs taken at various positions before and after 5 times of repeated folding under 1.5R condition.


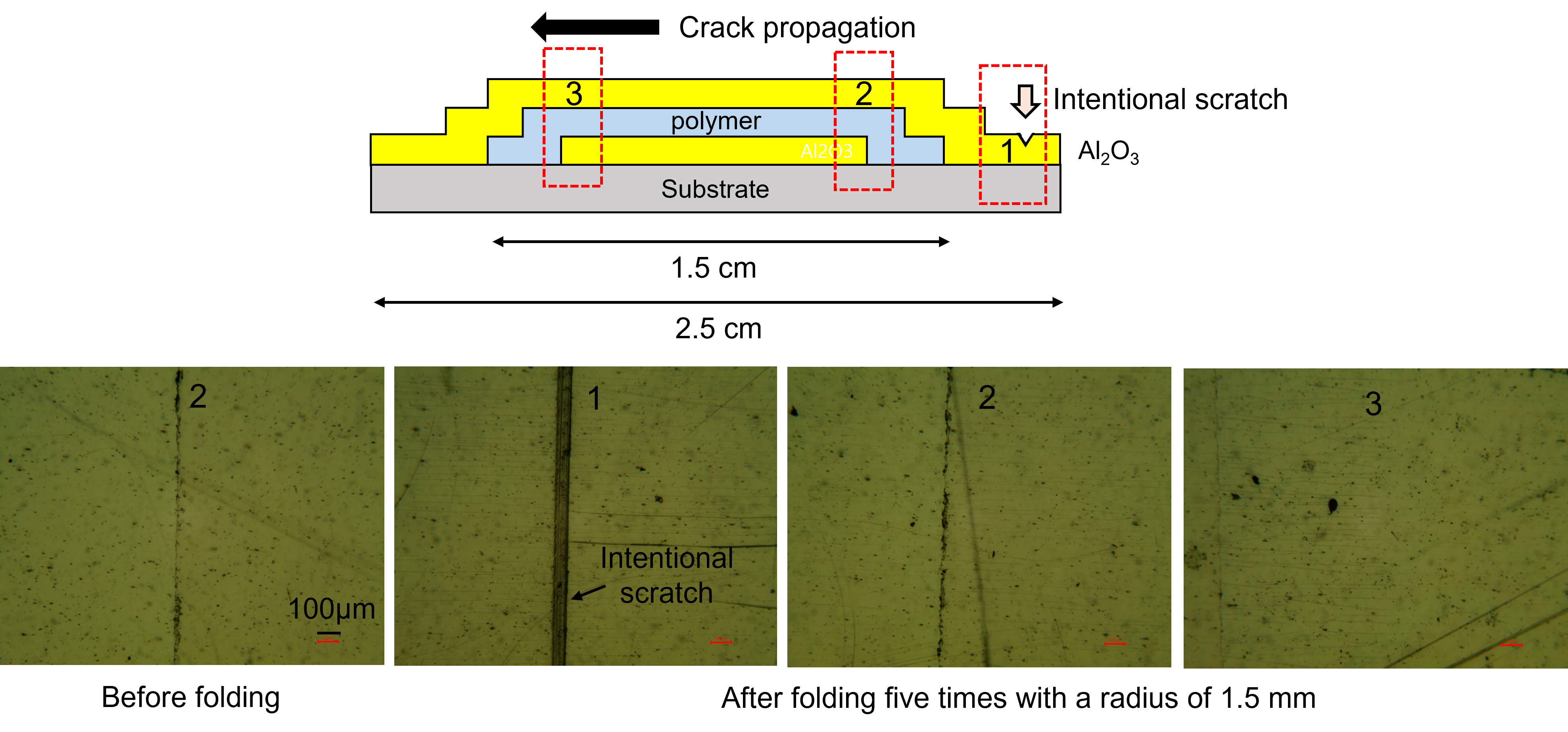


**Figure S5**. Cross-sectional schematic of the encapsulation structure in which two Al_2_O_3_ layers are separated, and surface photographs taken at various positions before and after 5 times of repeated folding under 1.5R condition.


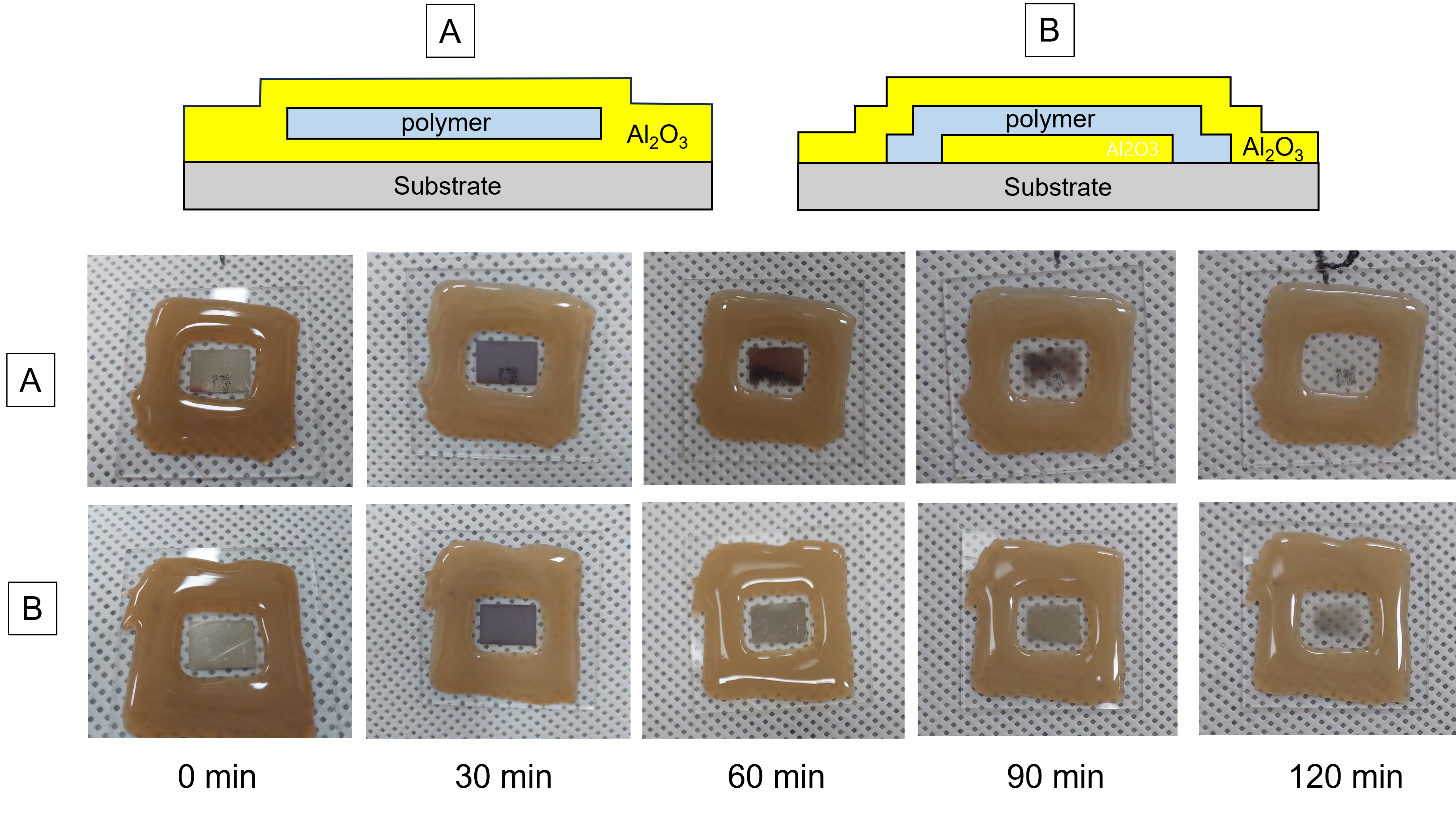


**Figure S6**. Optical Ca test results showing water permeation prevention properties of two encapsulation structures after folding deformation is applied.

**Table S1**. Water contact angles and surface energies of Si, FOTS/Si, and Al_2_O_3_ surfaces.

| Surface | θ_water_  (degree) | θ_diidomethane_  (degree) | γ_s_^Dispersive^  (mJ/m^2^) | γ_s_^Polar^  (mJ/m^2^) | Surface energy  γ_s_=γ_s_^Dispersive^+γ_s_^Polar^ |
| --- | --- | --- | --- | --- | --- |
| Si | 44.30 | 46.40 | 36.26 | 21.40 | 57.66 |
| FOTS/Si | 110.5 | 87.80 | 13.69 | 0.460 | 14.16 |
| Al_2_O_3_ | 17.20 | 36.00 | 41.56 | 31.20 | 72.76 |
